# Development of the Perth Adolescent Worry Scale (PAWS)

**DOI:** 10.1007/s10802-021-00853-6

**Published:** 2021-08-20

**Authors:** Simon C. Hunter, Stephen Houghton, Michael Kyron, David Lawrence, Andrew C. Page, Wai Chen, Leslie Macqueen

**Affiliations:** 1 Department of Psychology, Glasgow Caledonian University, Glasgow, UK; 2 Graduate School of Education, The University of Western Australia, Perth, WA Australia; 3 School of Psychological Sciences and Health, University of Strathclyde, Glasgow, UK; 4 School of Psychological Sciences, The University of Western Australia, Perth, Australia; 5 Curtin Medical School, Curtin University, Perth, Western Australia Australia; 6 Fiona Stanley Hospital, Murdoch, Perth, Western Australia Australia

**Keywords:** Worry, Adolescence, Peers, Academic Success

## Abstract

Tools to assess worry among adolescents exist but do not capture the content of worries. This study reports on the development of a brief, psychometrically sound measure of worry for use with adolescents. Phase 1 involved identification of 27 potential items from existing instruments as well as item generation identified in interviews with students, teachers, school psychologists, and parents. In Phase 2, the candidate items were completed by 835 Australian adolescents (317 males, 508 females, 10 unspecified; Mean age = 13.55, SD = 1.31) from Grades 5 to 10. These data were randomly split in half, and an exploratory factor analysis on the first half identified a two-factor solution with 12 items: Peer Relationships (6 items) and Academic Success and the Future (6 items). On the second half of the data, confirmatory factor analyses supported the factor structure and supported strong invariance across age, socioeconomic status, and presence/absence of a diagnosed neurodevelopmental disorder. Weak invariance was evident across sex. Differences across groups are reported as are correlations with indicators of psychological wellbeing. In conclusion, the Perth Adolescent Worry Scale provides both applied professionals and researchers with a short, easy-to-administer, and psychometrically strong instrument to evaluate adolescents’ everyday worries.

**Supplementary Information:**

The online version contains supplementary material available at 10.1007/s10802-021-00853-6.

Worry is a repetitive, future-oriented, and negatively valenced cognitive activity, with worry content often including how one might prevent or cope with negative future events and experiences (Borkovec et al., 2004; Mennin et al., 2005; Newman & Llera, 2011; Watkins et al., 2005). It is a key cognitive indicator of, and unifying process across, all anxiety disorders (Barlow, 2002; Berenbaum, 2010; Olatunji et al., 2010). Avoidance models of worry propose that worry is a short-term emotion regulation strategy which avoids more intense emotions by focusing attention on this less physiologically arousing cognitive activity (Behar et al., 2009; Newman & Llera, 2011). Worry may be used to prolong and maintain a chronic, negative emotional state to avoid an unexpected emotional shift from positive to negative emotion (Newman & Llera, 2011).

During adolescence, worry tends to be experienced as a normative and common thought process, with adolescents typically reporting multiple worries per day that often wax and wane with the everyday environmental pressures from school, peers, and family (Arbel et al., 2017). Generally, worries involve issues relating to school, interpersonal and social problems, relationships, health, achievement, social acceptance, psychological well‐being, exams, appearance, the future, upcoming stressful events, and money problems (Arbel et al., 2018; Hunter et al., 2014; Olatunji et al., 2010; Songco et al., 2020). Young adults tend to report greater levels of worry regarding academic performance and finances (Brzezinski et al., 2018). Generally, females report higher levels and greater frequencies of worry than males (Arbel et al., 2018; Caes et al., 2016).

The significant cognitive, social, and physiological changes that take place during adolescence can impact on the development of worry (Copeland et al., 2014) such that it becomes a prominent cognitive process in everyday functioning, particularly as these worries become more elaborate and abstract (e.g., Arbel et al., 2018; Laugesen et al., 2003; Muris et al., 2002). Whether worrying about the consequences of a past event, or anticipating the outcome of a future stressful event, emotional, cognitive, and physiological responses can become prolonged and exacerbated (Brosschot et al., 2006; Verkuil et al., 2010). For 25% of adolescents, worry reaches intense and uncontrollable levels (Arbel et al., 2018; Fournier et al., 1996). If left untreated, it can become pathological and lead to significant distress, anxiety disorders, mood disorders, insomnia, absence from school, withdrawal from social activities, and poor academic functioning (Albano & Hack, 2004; Purdon & Harrington, 2006). The health care and educational costs associated with worry are high (see Schroder et al., 2019). Therefore, quick, routine assessment of problematic worry would be immensely beneficial in terms of understanding the primary sources of worry among adolescents and ways in which to intervene.

Worries are positively correlated with measures of depression among young people (Boelen et al., 2010; Zimmer-Gembeck et al., 2010) and evidence with adult populations indicates that worry is correlated with depression even after controlling for anxiety (Parmentier et al., 2019; Swee et al., 2019). Thus, one measure of depression and one of internalization are utilised in the current study to establish convergent reliability. Worries often relate to behavioural competence and social evaluation (La Greca & Lopez, 1998; Vasey et al., 1994) and young people’s peer-victimization is positively correlated with worry (Schmidt & Bagwell, 2007). Recognizing this, the current study also used measures of loneliness and prosocial behaviour as indices of convergent validity. It was anticipated that worry would be positively correlated with scores on all measures, except for prosocial behaviour where a negative relationship was expected. Finally, evidence relating to the association between worry and aggression / disruptive behavior is less clear, with some authors reporting significant effects (Robertson et al., 2018; Whiting et al., 2014), some not (Blain-Arcaro & Vaillancourt, 2016), and some reporting effects only for youth with ASD (Bos et al., 2018). This being the case, an exploratory analysis involved examination of relationships between worry and a measure of externalising.

According to Maisel et al. (2019), it is imperative that simple and intuitive ways to manage the acute distress of negative thoughts in everyday life are forthcoming. However, the underlying mechanisms involved in worry may be complex. For example, adolescents with Neurodevelopmental Disorders (NDDs e.g., Specific Learning Disorders, Attention Deficit Hyperactivity Disorder, Intellectual Developmental Disorders, Communication Disorders, Autism Spectrum Disorder) may be more prone to cognitive biases that precede worry (see Schmidt & Vereenooghe, 2020) because of a tendency to interpret everyday ambiguous interpersonal stimuli, arising from other people’s behaviours or facial expressions, as threatening or negative (see Hiemstra et al., 2019). There is also a focussed body of research indicating that people with Autism Spectrum Disorder (ASD) may have unique worries (see Magiati et al., 2017, for a review) and that measures of parent-reported anxiety have different factor structures across populations with ASD, without ASD, and with Anxiety (Glod et al., 2017; Toscano et al., 2020a, b). With 15–20% of adolescents presenting with a NDD, prevalence rates rising (King-Dowling et al., 2019), and educational, psychosocial and health care costs for individuals with NDDs being significantly higher than that of their typically developing peers (Beckman et al., 2016; Sciberras et al., 2017; Wilkes et al., 2012), determining potential differences is clearly important.

In addition to supporting exploration of differences across NDD and non-NDD populations, measures of worry should be also designed to facilitate research on developmental differences across adolescence, across gender, and across different socioeconomic strata. Evidence exists that girls may worry more than boys on a range of worries (Lin et al., 2017; Orton, 1982; Silverman et al., 1995) except everyday events which boys worry more about (Hencker et al., 1995) and that youth in lower socioeconomic strata worry more than those in higher strata (Orton, 1982). In addition, older youth tend to report more worries about economic issues, academic performance, and physical appearance (Hencker et al., 1995; Simon & Ward, 1974; Weems et al., 2000). With such differences evident in the literature, it is important that measures of worry be invariant in their measurement, that is to say, that scale scores across these different groups can be trusted to be equivalent in meaning.

The most popular (and considered the gold standard) inventory of problematic worry used in research is the 16-item self-report Penn State Worry Questionnaire (PSWQ; Meyer et al., 1990). However, the factorial structure of the scale remains contentious with single factor, two-factor, and possible single higher order factor structures being proposed (see Berle et al., 2011). Item overlap and redundancy (e.g., ‘I worry all the time’ and ‘I am always worrying about something’) is also a limitation. Consequently, shorter versions of the PSWQ have been developed, including10-item (Yao et al., 2016), 8-item (Hopko et al., 2003), 5-item (Topper et al., 2014), and 3-item (“ultra‐brief”) (Berle et al., 2011) versions to capture global feelings of worry.

The psychometric properties of these shortened versions of the PSWQ suggest they all perform well as proxies for the full measures, and are suitable to measure pathological worry, especially in clinical practice settings (Berle et al., 2011; Topper et al., 2014). However, these measures tend to represent problematic worry as a unidimensional continuous construct and do not capture the *content* of the worry. Furthermore, they were administered to older adolescents, university students and adults, and in many cases on commencement or completion of treatment for a diagnosed anxiety disorder, and so may not be appropriate for the whole adolescent age spectrum.

A 14-item child version of the PSWQ also exists (The Penn-State Worry Questionnaire for Children, PSWQ-C; Chorpita et al., 1997). Adapted from the PSWQ, the PSWQ-C measures excessive, generalized, and uncontrollable worry. Studies with community and clinical samples aged 5 to 19 years (Chorpita et al., 1997; Esbjørn et al., 2013; Muris et al., 2001; Pestle et al., 2008) have indicated the PSWQ–C has either one or two factors. In studies where two factors are indicated, the psychometric evaluation has suggested that the three reverse-scored items should be removed, as they may be a statistical artefact rather than a meaningful construct. Although the PSWQ-C captures the essence of pathological worry it was derived from adult conceptualisations of worry and neither refers to modern issues faced by young people (e.g., adverse experiences with technology such as social media use or gaming) nor to other age-specific issues such as schooling (e.g., not doing well), social image, news about crime or attacks, or the environment.

Aside from the PSWQ and its derivatives, there also exists the Worry Scale (Perrin & Last, 1997), Arbel et al.’s (2018) daily worry scale, Forte et al.’s (2011) semi‐structured ‘worry’ interview, Orton’s (1982) Worries Inventory, Simon and Ward’s (1974) Worry List Questionnaires, and Lin et al.’s (2012, cited in Lin et al., 2017) Worry Tendency Questionnaire for Chinese youth. Each of these measures have their own weaknesses, for example being interview-based, being developed almost a century ago, or being based exclusively on diagnostic criteria contained in DSM III (American Psychiatric Association, 1980).

The lack of instrumentation highlights the need to develop a psychometrically sound, modern, and brief measure to assess worry among adolescents, one which allows for direct comparisons across key demographics. As highlighted by Songco et al. (2020), there is also a need to develop validated measures of worry that more appropriately reflect age relevant concepts for the assessment of worry in young people. Therefore, the current study sought to identifying candidate items with which to develop a brief, psychometrically sound measure to assess adolescent worry and, subsequently, to assess indices of validity and the extent to which it is invariant across gender, age, socio-educational advantage, and presence/absence of a diagnosed neurodevelopmental disorder.

## Method

### Phase 1: The Development of the Perth Adolescent Worry Scale (PAWS)

The first step was to review the relevant literature and the existing instruments to identify potential items for inclusion in the new instrument. To be included in the review, instruments had to have been used in research published in peer-reviewed journals and, where possible, include items applicable to adolescents. Six main instruments were identified: The PSWQ (Meyer et al., 1990), The PSWQ-C (Chorpita et al., 1997), The Child and Adolescent Worry Scale (Campbell & Rapee, 1994), The Worry Scale (Perrin & Last, 1997), Byrne et al.’s (2007) Adolescent Stress Questionnaire, and Arbel et al.’s (2018) Daily Worry Scale. In addition, Forte et al.’s (2011) semi‐structured ‘worry’ interview, which asked 17–20 year olds with and without an Intellectual Disability to identify their four most salient worries from a series of 12 photographs was reviewed.

A panel of four individuals with between 5 and 30 years of expertise in child and adolescent psychology, and measurement and assessment (one had recently completed an undergraduate degree and had worked with children and adolescents; one had recently completed a PhD in Clinical Psychology and had substantial experience working in a clinic which primarily focuses on anxiety disorders in young people; two other panel members were senior academic researchers with over 20 years of clinical and school experience and currently leading large scale projects in adolescent psychopathology) reached consensus that no items in the Penn State Worry Questionnaires were suitable for inclusion because their focus was on how *often* a person worried *generally* (e.g., “many situations made me worry”; “I knew I shouldn’t worry about things, but I just couldn’t help it”; “I was always worrying about something”), rather than identifying specific areas or behaviours of worry. Conversely, Arbel et al. (2018) provided areas for adolescent worries pertaining to appearance, money, school grades, boyfriend/girlfriend, being bullied, personal health, family, and friends. However, although Arbel et al. (2018) recognised the multidimensional nature of adolescent worry and included items that were specific to areas of worry, many items were presented in a general manner (e.g., Today how much did you worry about “something you heard”; “your health”) or covered multiple behaviours in a single item (e.g., “your own health or health habits e.g., smoking, alcohol, drugs, eating”; “sexually transmitted diseases or pregnancy”; “sports, music dance or drama performance”). In addition, the sample comprised of “late adolescents”. Nevertheless, 11 possible items were generated: Five were drawn from the Adolescent Stress Questionnaire (Byrne et al., 2007), three from Arbel et al.’s (2018) Daily Worry Scale, and one from each of Perrin and Last’s (1997) Worry Scale, Forte et al.’s (2011) worry interview, and Brzezinski et al.’s (2018) Social Worry Scale.

To develop a comprehensive coverage of worry in adolescents and to generate potential new items or areas, eight separate focus groups comprising three to five participants were conducted with randomly selected students (n = 36) (54% male, age range 10 to 16 years; equally distributed across school grades 5 [age 10 years], 7 [12–13 years], 9 [14–15 years] and 11 [15–16 years]). Of these, eight had neurodevelopmental disorders. In addition, four focus groups were conducted with parents/Guardians (n = 16) (80% mothers, age range 34–50 years) and five focus groups with teachers (n = 16) (3 females and 2 males from one non-government school, and 4 females and 7 males from three government schools; teaching experience ranging from 2 to 25 years). The number of focus groups was based on Guest et al.’s (2017) thematic analysis of 40 focus groups on health-seeking behaviours which recommended that 80% or more of all themes are discoverable within two to three focus groups and 90% within three to six focus groups. The number of participants within each focus group ranged from three to five to maintain engagement and interaction (Willig, 2001).

Individual interviews were also conducted with eight school psychologists (2 from one non-government school and 6 from government schools; experience ranging from 2 to 18 years) from schools participating in the validation study. Within the interviews, participants were asked what young people worry about in general, how often they worry about these, and how intense the worries tended to be. The moderator conducting the interviews continued with the questions until no new information was being offered by participants.

The interviews and focus groups were part of a larger longitudinal study examining trajectories of mental health in adolescents and the only potential participants who declined to be involved when invited were parents, the primary reason offered being they were at work. Each focus group was carried out by one of two moderators, each of whom had extensive experience (over 15 years) of the technique. Moderators worked from a script though novel issues were explored. The sessions lasted for between 25–45 min.

The four participating schools were located in a range of socio-economic status areas as indicated by their Index of Community Socio-Educational Advantage (ICSEA). ICSEA is set at an average of 1000 (SD = 100) and the higher the ICSEA value, the higher the level of educational advantage of students who go to this school (and vice versa). The non-government secondary school had the highest ICSEA value (1191), the rural located government secondary school recorded an ICSEA value of 904, and the two metropolitan government schools had values of 939 and 980.

Prior to the interviews being conducted, permission was obtained from the Human Research Ethics Committee of the University of Western Australia and the participating schools. Informed written consent was also obtained from the parents and participating students. The moderator met participants at pre-arranged times and all interviews were conducted in a room set aside within the respective schools for this purpose.

Thematic analysis was used to identify, analyze, organize, describe, and report themes found within the interviews (Braun & Clarke, 2006) and to identify patterns of meaning that repeatedly emerged. This involved initial production of codes from the data, sorting and collating all the potentially relevant coded data extracts into themes, reviewing the coded data extracts for each theme to consider whether they appear to form a coherent pattern, and determining what aspect of the data each theme captures. SH, MK, DL and LM decided the final set of items following discussions about the themes and content that emerged from the interviews. The same researchers discussed similar items to determine their closeness to each other and therefore whether they should be retained or not. The major finding from the interviews with adolescents, parents, teachers and school psychologists was that young people experience multiple worries throughout the day (from waking to going to sleep at night) about peer friendships and interactions, school success and failure, challenges to their own mental health, and their appearance and social image. Social media was a major issue in the generation of everyday worries. Early adolescents and adolescents of both sexes tended to report the same types of worries, although females tended to interpret some worries (e.g., body image) more intensely.

From the interviews 16 new items were generated for the new scale, resulting in 27 potential items in total (see Table 1). All 27 items were reviewed to be in a self-report appropriate format. The readability levels of the newly developed candidate 27-items for the worry scale was measured using The Flesch-Kincaid Grade Level (i.e., the number of years of education required to understand a standard reading passage) and The Flesch Reading Ease (i.e., the difficulty level of reading a normal reading passage) (Flesch, 1948; Microsoft Corporation, 2010). The Worry scale was considered appropriate, and comprehensible and easy (reading ease = 83.3) for Australian school students enrolled in Grades 5/6 (Flesch-Kincaid Grade Level; age 10 years and above).

### Phase 2: Validation of the Perth Adolescent Worry Scale

#### Participants and Settings

The total sample comprised of 835 adolescents (317 males, 508 females, 10 unspecified; Mean age = 13.55, SD = 1.31) from Grades 5 (11–12 years of age: N = 98, 54 males, 44 females), 6 (11–13 years of age: N = 50, 26 males, 23 females, 1 unspecified), 7 (12–15 years of age: N = 245, 76 males, 166 females, 3 unspecified), 8 (13–15 years of age: N = 201, 74 males, 123 females, 4 unspecified), and 9 (13–16 years of age: N = 236, 84 males, 150 females, 2 unspecified). Students in grades 10 and 11 were not invited to take part, but two students mistakenly reported that they were in either grade 10 (16 years of age: N = 1 male) or 11 (16 years of age: N = 1 male). Two participants did not report their age and one did not report their Grade level.

Of these students, 92 (11–16 years of age: 44 males, 46 females, 2 unspecified) were determined to have been clinically diagnosed by a paediatrician or child psychiatrist as meeting DSM-IV-TR (American Psychiatric Association, 2000) or DSM 5 (American Psychiatric Association, 2013) criteria for a neurodevelopmental disorder (e.g., Attention Deficit Hyperactivity Disorder, Autism Spectrum Disorder, Specific Learning Disorder). This was determined in two ways. First, students were asked to self-report if they had a diagnosis of ADHD, ASD, or SLD from a list provided in the survey. Once the survey was completed in each school, the School Psychologist, Principal, and/or Year Coordinator confirmed the accuracy of this via school records. In addition, School Psychologists, Principals, and/or Year Coordinators reviewed the list of participants from each year group and identified students with diagnoses, subsequently confirmed via official school records. At no point did school staff report, or confirm, *specific* diagnoses. All young people with an NDD diagnosis were attending mainstream High schools, the same schools as the non-NDD group.

Participants were recruited from 11 randomly selected secondary schools, of which eight were state government schools, and three were non-government schools. The ICSEA for these schools showed they were located across a range of socio-economic areas. Two of the three non-government secondary schools had the highest ICSEA values (1191, 1068), while the third had an ICSEA value of 986. Of the state government secondary schools seven recorded ICSEA values ranging from 829 to 980, and one was 1003.

## Measures

The 27 candidate items for the Perth Adolescent Worry Scale (PAWS) required participants to self-report how often the issue was worried about (0 = “Never”, 1 = “Sometimes”, 2 = “Often”, 3 = “Always”) and how much worry it caused the respondent (0 = “Not at all”, 1 = “A little bit”, 2 = “Somewhat”, 3 = “A lot”). Individual items for use in the exploratory and confirmatory factor analyses used a set of items which were created by combining these two items for each worry by multiplying the relevant responses. For example, the responses assessing frequency of worry and degree of worry for the item “Exams or tests” were combined by creating the product of these two estimates (creating a 0–9 scale for each item used in the factor analyses).

The Children’s Depression Inventory 2 (self-report short version; CDI:SR[S]2; Kovacs, 2004) is a brief self-report assessment of cognitive, affective and behavioural symptoms of depression in children and adolescents aged 7–17 years. The CDI:SR[S]2 comprises of 12 items, each with three separate sentence response options that describe participants’ feelings and ideas over the past two weeks. Each item is measured on a 3-point Likert scale, with higher scores indicating poorer outcomes (e.g., 0 = I am sad once in a while, 1 = I am sad many times, 2 = I am sad all the time). Total raw scores were converted to a standardised T score (mean = 50, SD = 10). The CDI:SR[S]2 has demonstrated good reliability (α = 0.77-0.85 across different age and gender groups; test–retest estimates from 0.76-0.92: Kovacs, 2004), and discriminant and convergent validity with other measures of depression, anxiety, self-competence, self-concept, and locus of control (Doerfler et al., 1988). Good internal reliability has been reported in neurologically diverse samples of Australian youth (α = 0.86: Houghton et al., 2020). In the present study, the CDI:SR[S]2 had good internal reliability (α = 0.85).

The 24-item Perth Aloneness Scale (PALs; Houghton et al., 2014) uses a six-point Likert scale (1 = Never, to 6 = Always) to measure four distinct aspects of loneliness: isolation (i.e., having few friends or believing that there is no one around offering support); friendship related loneliness (i.e., having reliable, trustworthy supportive friends); negative attitude to solitude (i.e., negative aspects of being alone such as time dragging, isolation); and positive attitude to solitude (i.e., positive aspects and benefits of being alone such as relaxing, happiness). Higher scores for friendship related loneliness indicate greater quality of friendships, while higher scores for isolation indicate higher levels of isolation. The scale has shown strong psychometric properties in prior research (Houghton et al., 2014, 2016, 2020). In the present study, each factor demonstrated acceptable-excellent internal reliability (friendship related loneliness α = 0.90; isolation α = 0.86; positive attitude to solitude α = 0.87; negative attitude to solitude α = 0.79).

The 25-item self-report Strengths and Difficulties Questionnaire (SDQ-SR; Goodman, 1997) is a screening measure to indicate clinical levels of symptoms in the present, or risk for them in the future. The SDQ-SR’s 25 items are equally spread across five subscales, four of which measure mental health (*Emotional Symptoms*, *Peer Relationship Problems*, *Conduct Problems,* and *Hyperactivity-Inattention*) and one that measures strengths (*Pro-Social Behaviour).* Participants respond to items as “not true”, “somewhat true”, or “certainly true”. Although the SDQ is designed to produce five subscales (i.e., emotional problems, conduct problems, hyperactivity, peer relationship problems, and prosocial behavior), a three-subscale division is recommended when assessing non-clinical populations (see Carroll et al., 2020; Goodman et al., 2010). These three subscales comprise an internalizing subscale (emotional problems and peer problems summed), an externalizing subscale (conduct problems and hyperactivity summed), and a prosocial scale with internal reliability reported to be 0.66, 0.76, and 0.66 respectively (Goodman et al., 2010). In the present study, each demonstrated acceptable-excellent internal reliability (internalizing α = 0.76; externalizing α = 0.79; prosocial α = 0.71).

## Procedure

The Human Research Ethics Committees of The University of Western Australia, the Western Australian Department of Education and the Principals of the non-government schools granted permission to conduct this research. Information sheets were sent to the parents of students in school grades 5 to 9 (11 – 16 years of age) explaining the research along with consent forms. Informed consent was obtained from individual participants included in the study. The measures were completed by adolescents via an online survey during regular school hours and a teacher and/or School Psychologist was present to support any students who had difficulty understanding any items. Each participant received a unique identification code, which allowed them to log on to the survey. This unique code ensured that all information provided was confidential. To ensure measures were administered consistently across schools one teacher in each of the schools volunteered to be responsible for liaising with the researchers and administering the survey. Written instructions regarding administration procedures were provided to all of these teachers along with verbal instructions. The electronic survey remained open for approximately four weeks.

## Analytical Approach

The sample was randomly split into two halves. An exploratory factor analysis was conducted on one half of the sample. A confirmatory factor analysis was fit to the second half to confirm the fit of the factor structure identified in the EFA. The entire dataset was then used to test for invariance of the factor structure across age, gender, socio-economic status and NDD status. We did not use change in chi-square as an indicator of invariance because of its documented sensitivity to sample size. Rather, we used change in CFI (∆CFI) as one indicator of invariance (∆CFI > -0.01 indicates violation of invariance) and whether the invariance model’s 90% RMSEA confidence intervals included the RMSEA point estimate of the unconstrained model. For the purposes of analyses, the participants were grouped into two age groups, namely early adolescents 11 -13 years (n = 384, 153 males, 228 females, 3 unspecified) and adolescents 14 + years (n = 449, 163 males, 279 females 7 unspecified). Note that two participants were excluded from this analysis as they failed to report age.

## Results

There were negligible levels of missing data (0% on individual worry items; 0% on depression scale scores; 0% on loneliness attitudes scale scores; 0.1% on both the Friendship and Isolation loneliness scores; 1.3% on Internalizing SDQ scores; 0.8% on Externalizing SDQ scores; and 0.7% on Prosocial SDQ scores). Since these levels are well below 5%, no action was taken to address them (Tabachnick & Fidell, 2007).

An exploratory factor analysis (EFA) was conducted on the full set of 27 items, using scores which were the product of the frequency and severity estimates for each. The EFA was conducted using a random split of the data set (n = 418). Using SPSS25, and following the recommendations of Howard (2016), we conducted an EFA with a direct oblimin rotation (with a delta of zero) as we expected that any identified factors would be correlated. Six factors had eigenvalues over 1, and 30.7% of the variance was accounted for by a first factor, 7.9% by the second, 5.7% by a third, 5.1%, by the fourth, 4.3% by the fifth, and 3.8% by the sixth factor. Factor loadings are reported in Table 1, alongside descriptive statistics for the original items. The scree plot suggested that a 2-to-3 factor solution was optimal. To establish which items were appropriate to which factors, Howard’s (2016) 0.40–0.30–0.20 rule was followed. Examination of the rotated pattern matrix indicated that a 2 factor solution was optimal, with six items relating to Peer Relationships and six items relating to Academic Success and the Future.

Following the EFA, we assessed the fit of the factor structure using confirmatory factor analysis (CFA) using AMOS26. This analysis used the second half of the data set (n = 417). Goodness of fit was assessed with the Comparative Fit Index (CFI: above 0.95 indicates good fit, above 0.90 indicates adequate fit) and the root mean-square error of approximation (RMSEA: 0.05 or less indicates good fit, 0.08 or less indicates adequate fit). The model was an acceptable fit to the data: *χ*^*2*^ (df = 53) = 222.03, *p* < 0.001; CFI = 0.928; RMSEA = 0.088 (90% Confidence Interval = 0.076, 0.100). The standardised factor loadings are reported in Table 2. Cronbach’s alpha indicated satisfactory internal reliability for both subscales (α_Peer_ = 0.83; α_Academic_ = 0.88). The final version of the scale is included in the Appendix.

The invariance of the identified factor structure across sex (male, female), age (11–13, 14 +), ICSEA (bottom 25%, middle 50%, top 25%), and NDD status (NDD, Non-NDD) was assessed using the entire data set. To establish invariance, three models were compared for each invariance test. The first model was an unconstrained model where the same factor structure was present for the competing groups (e.g., males and females) but there were no further model constraints. The second model (testing weak invariance) added constraints upon the factor loadings. Finally, the third model (testing strong invariance) also constrained indicator intercepts to be equal. Strong factorial invariance indicates that slopes and intercepts are equal across groups, supporting the assertion that factor scores are comparable across groups (Schumacker & Lomax, 2004). The change in chi-square was not used to assess invariance due to its documented sensitivity to sample size (Schumacker & Lomax, 2004). Rather, we used change in CFI, where invariance is violated in cases with a drop of -0.01 or more and where the invariance model’s 90% RMSEA confidence intervals included the RMSEA point estimate of the unconstrained model (Schumacker & Lomax, 2004).

The results of the four tests of invariance are reported in Tables 3 (for sex and age) and 4 (for NDD status and ICSEA). Strong invariance was supported for NDD status. Weak invariance was supported across ICSEA, Sex and Age. We also argue for strong invariance across ICSEA and Age as the point estimate of the unconstrained model was contained with the 90% RMSEA confidence intervals of the strong invariance model while the reduction in CFI was 0.014 and 0.018 respectively when moving from weak to strong invariance.

We next calculated scale scores for the complete data set using factor score weights provided in AMOS (see Table 2). The mean factor scores are reported in Table 5, shown by ICSEA level, NDD status, and age since these three variables all demonstrated strong invariance. Scores across both scales were significantly lower for participants diagnosed with an NDD than those without (Academic Success and Future: *t* (833) = 4.73, *p* < 0.001, Cohen’s *d* = 0.56; Peer Relationships: *t* (833) = 3.06, *p* = 0.002, Cohen’s *d* = 0.40). Young participants reported significantly lower Academic Success and the Future scores (*t* (831) = -3.20, *p* < 0.001, Cohen’s *d* = 0.23) and significantly higher Peer Relationships worries (*t* (831) = 2.77, *p* = 0.006, Cohen’s *d* = 0.19). Subscale scores did not differ by ICSEA level (*p*’s > 0.420).

As shown in Table 6, Peer Relationships factor scores were significantly and positively correlated with: depression, the Internalising and Externalising subscales of the SDQ, and two PALS subscales (Isolation, Negative Attitudes). Peer Relationships factor scores were also significantly and negatively correlated with the PALS Friendship subscale. The Peer Relationships factor scores were not correlated with the SDQ subscale assessing Prosocial behavior nor PALS subscale scores assessing Positive Attitudes.

Also shown in Table 6 are correlations between the factor scores for Academic Success and the Future and all other variables. All correlations followed the same pattern of significance and directionality as for the Peer Relationships factor, except that Academic Success and the Future factor scores are significantly and positively correlated with Positive Attitudes toward loneliness.

## Discussion

This study presents the development of a brief and psychometrically sound measure for the assessment of adolescent worry, the Perth Adolescent Worry Scale (PAWS). To generate an appropriate range of candidate items, examples were drawn from related measures and combined with the results of semi-structured interviews conducted with students, parents, teachers, and school psychologists. The 27 candidate items were subsequently presented as a single scale to a sample of 835 adolescents ranging from 11 to 16 years old from across a range of socio-economic areas and including young people both with and without clinically diagnosed neurodevelopmental disorders. This resulted in identification of a two-factor solution, with six items relating to Peer Relationships and six items relating to Academic Success and the Future.

Both subscales demonstrated good internal reliability, and strong measurement invariance was identified when comparing young people with neurodevelopmental disorders to those without such disorders. This is a clear strength of the scale and suggests that it can be used to appropriately compare the worries of young people with a range of neurodevelopmental disorders to those without. This can potentially enhance the empirical base underpinning understanding of these issues in sample of young people with conditions such as ADHD, autism, or learning disorders. More speculatively, and subject to further investigation of its use with this diverse group, the measure may also be helpful for practioners. For example, sleep problems frequently occur among children with NDDs (see Tan-MacNeill & BAe, 2014), are known to be associated with worry (O’Kearney & Pech, 2014), and so formulation work during intervention design (Phillips et al., 2020) may benefit from use of the PAWS.

In our sample, scores on the two subscales were significantly lower among young people with NDDs than those without NDDs. This is in contrast to work indicating that children and young people with NDDs typically experience comorbid anxiety (see Hansen et al., 2018) which might raise the expectation of higher, not lower, levels of worry. This may reflect reports that highly worried individuals do not always meet diagnostic criteria for anxiety disorders and that additional risk factors require to be present to convert worry into problematic anxiety (Borkovec & Inz, 1990; Ruscio & Borkovec, 2004). It may also be the case that youth diagnosed with ADHD were over-represented in our sample and, if this is the case, then low scores may reflect poor emotional awareness (Factor et al., 2016). Finally, there may be nuances around the concept of worry that are deserving of future research attention. Specifically, anxiety and stress are not clearly differentiated concepts in mid-to-late childhood (Szabo & Lovibond, 2006) and our results may be picking up similar issues concerning what children with NDDs worry about and how much they report worrying about those issues that concern them.

There was also support for strong invariance across the two age groups (11–13, 14 +) and across low, middle, and high ICSEA levels, and weak invariance for Sex. Demonstrating strong measurement invariance permits unambiguous interpretation of analyses investigating between-group difference (Cheung & Rensvold, 2002). This indicates that the PAWS is appropriate for research investigating young people’s worries because the scores have equivalent meaning for young people across the groups (e.g., for those aged 11–13 and those aged 14 +) where strong invariance is supported. Scores on the Academic Success and the Future scale were significantly lower for younger than for older students. This pattern was reversed for the Peer Relationships scores, where younger students’ scores were significantly higher than those reported by older students. There was no statistically significant difference on either subscale according to ICSEA level. Since only weak invariance was supported for the scores reported by boys and by girls, these were not compared. A lack of strong invariance does not invalidate the measure’s utility for assessing worry among boys or girls, but rather it clarifies that it is not appropriate for users of the scale to directly compare boys’ and girls’ scores.

We also examined the correlations between each PAWS subscale score and symptoms of depression as assessed by the CDI:SR[S]2 (Kovacs, 2004); the Internalising, Externalising, and Prosocial subscales of the SDQ (Goodman et al., 2010); and the Isolation Loneliness, Friendship Loneliness, Positive Attitudes (towards loneliness), and Negative Attitudes (towards loneliness) sub-scales of the PALs (Houghton et al., 2014). These indicate that worries concerning Peer Relationships positively correlate with depression, the Internalising and Externalising subscales of the SDQ, and two PALS subscales (Isolation and Negative Attitudes scales). Worries about Peer Relationships also negatively correlate with the PALS Friendship subscale. Clearly, worries about social and interpersonal relationships are important for young people and these co-occur with wider issues concerning psychological wellbeing. The PAWS provides researchers with a measure which can be used to unpack theoretically important issues of causality among these outcomes in future empirical work, though future psychometric data is welcome.

Worries about Peer Relationships were correlated with neither the PALS subscale scores assessing Positive Attitudes nor the SDQ subscale assessing Prosocial behavior. Though we cannot determine causality, there is research supporting the beneficial effects of prosocial behavior on friendship formation, quality and reciprocity (Cillessen et al., 2005; Dirks et al., 2018; Menisini, 1997). Thus, the absence of an association between worries concerning peer relationships and prosocial behavior may indicate that young people’s efforts to engage in positive ways with their peers do not prevent them continuing to worry about those relationships. More generally though, the degree to which worries reflect *potential* friendship difficulties and the extent to which they reflect *likely* friendship difficulties is something that cognitive bias modification for interpretation approaches (Joormann et al., 2015) might usefully attempt to address given their focus on accurate interpretation of one’s environment.

Worries about Academic Success and the Future followed the same pattern of significance and directionality of association with wellbeing indicators as the Peer Relationships factor, except that this scale is also positively associated with Positive Attitudes toward loneliness. Clearly, worries about schoolwork and the future are important for young people and these co-occur with wider issues concerning psychological wellbeing. While worries such as these are likely to be both normative and realistic, it is also important that they do not become debilitating or overwhelming for young people.

## Strengths and Limitations

The development of the PAWS has evidenced many strengths including a rigorous procedure for identifying candidate items, assessment of measurement invariance across important characteristics, and investigation of group differences and correlations with important indicators of psychological wellbeing. In addition, the inclusion of factor scores in our results allows other researchers who do not use, or do not have access to, structural equation modeling software to create their own factor scores that are appropriately weighted.

However, our approach is not without limitations and these include a lack of external validation that may be achieved in future by seeking the views of outside agents. For example, parent/guardian-reports and peer-reports could be compared to young people’s self-reports, though researchers have to be cognizant of the fact that worries may not be accessible to external factors (i.e. a young person may hide their worries from others). Another weakness of the development work reported here is that there is not yet any report of the stability of scores over time, nor in populations of young people with NDDs which are more severe than those of our participants, and future work should seek to achieve this. Related to this point is the recognition that NDDs represent a heterogeneous range of conditions and as such future work should seek to validate the PAWS separately within specific diagnostic subgroups. Finally, there exists evidence that worry may be culture specific (Lewis-Fernández et al., 2010; Silverman et al., 1995) and future development work should include cross-cultural validation of the PAWS.

A last point to note is that it may be helpful for future research to examine convergent validity with measures of anxiety. While it is important to retain an awareness that worry and anxiety are not interchangeable it may reasonably be expected that assessments of worry with align to a significant degree with assessments of anxiety. Similarly, it may be advantageous to examine correlations with other worry measures; the scope to do so here was limited because of our item identification strategy which included surveying the very measures that have the clearest potential to satisfy this aim (e.g., the Penn State Worry Questionnaire: Meyer et al., 1990).

## Conclusions

0The present study contributes to a limited body of research by reporting on the development of the PAWS, a promising measure for assessing worries surrounding both peers and academic issues that adolescents experience. While further validation work is still required, the PAWS offers psychologists, researchers and allied clinicians with a potential new, short, easy-to-administer instrument with which to evaluate adolescents’ everyday worries. As such, it also has the potential to provide an additional measure for evaluating strategies implemented to address problems such as anxiety. Future work should also seek to determine causality between adjustment indices and these worries.

**Table 1:** Factor Loadings* from Exploratory Factor Analysis (*n*=418) and Descriptive Statistics (*N*=835) for All Items

Item	Factor						Mean (SD) Worry	
	1	2	3	4	5	6	Frequency	Amount
Having a boyfriend or girlfriend (i.e. finding or maintaining a relationship)^a^	0.55						0.79 (0.98)	0.82 (1.01)
People talking about you online^a^	0.71						0.75 (0.97)	0.77 (0.97)
Not getting enough free time			0.73				1.09 (0.98)	1.05 (0.97)
Keeping up with school work^b^		-0.78					1.54 (1.00)	1.57 (1.03)
News of crime or attacks					0.84		0.80 (0.91)	0.87 (0.95)
Arguments at home						0.47	1.19 (1.03)	1.26 (1.07)
Not having enough time to game (e.g. PC or console)			0.86				0.62 (0.94)	0.61 (0.94)
The way you look	0.37			-0.32		0.42	1.41 (1.09)	1.40 (1.08)
Your social media profile (e.g. Instagram)				-0.67			0.59 (0.83)	0.58 (0.83)
What you will be doing when you finish high school^b^		-0.73					1.58 (1.04)	1.57 (1.07)
Letting your parents down^b^		-0.54					1.61 (1.11)	1.67 (1.12)
The environment (e.g. climate change)					0.69		1.23 (0.99)	1.26 (1.03)
Not having enough money to buy the things you want			0.41	-0.35			1.12 (1.01)	1.07 (0.98)
Your mental or physical health						0.53	1.31 (1.06)	1.35 (1.07)
Exams or tests^b^		-0.79					1.86 (1.04)	1.89 (1.05)
How much you eat						0.83	1.16 (1.03)	1.04 (0.97)
Not doing well in school^b^		-0.76					1.64 (0.98)	1.78 (1.04)
Fitting in with other students at school^a^	0.69						1.12 (0.99)	1.12 (1.01)
Not having enough time to social network (e.g. WhatsApp, messenger)			0.41	-0.39			0.58 (0.83)	0.55 (0.80)
The health of your family members		-0.36			0.38		1.74 (1.02)	1.17 (1.03)
Being bullied at school^a^	0.72						0.62 (0.90)	0.72 (0.98)
Falling out with your friends^a^	0.68						1.12 (0.99)	1.23 (1.04)
Missing out on social events (e.g. parties, sleep overs)	0.39			-0.37			1.01 (0.94)	1.01 (0.96)
Your future^b^		-0.73					1.71 (1.03)	1.69 (1.06)
Teachers treating you unfairly				0.38		0.40	0.92 (0.98)	0.90 (0.99)
Not being good at sport	0.39						0.81 (0.98	0.77 (0.96)
Being judged by your friends^a^	0.70						1.14 (1.04)	1.13 (1.06)

*Factor loadings below .30 not shown

^a^Items identified for Peer Relationships Sub-Scale

^b^Items identified for Academic Success and the Future Sub-Scale

**Table 2:** Factor Loadings from Confirmatory Factor Analysis (*n*=417) (and Factor Score Weights, *N*=835)

Item	Academic Success and the Future	Peer Relationships
Exams or tests	0.76 (.126)	
Keep up with school work	0.74 (.149)	
Not doing well in school	0.80 (.179)	
Your future	0.78 (.156)	
What you will be doing when you finish High school	0.71 (.141)	
Letting your parents down	0.77 (.119)	
People talking about you online		0.75 (.107)
Fitting in with other students at school		0.60 (.076)
Falling out with your friends		0.70 (.105)
Being judged by your friends		0.86 (.179)
Being bullied at school		0.64 (.091)
Having a boyfriend or girlfriend		0.45 (.043)

**Table 3:** Fit Indices Used for Assessing Factor Invariance Across Sex and Age (*N*=835)

Model	CFI		∆CFI (vs. preceding model)		RMSEA (90% CI)	
	Sex	Age	Sex	Age	Sex	Age
Model 1 (Unconstrained)	0.940	0.943	-	-	0.053 (0.047, 0.060)	0.054 (0.048, 0.060)
Model 2 (Weak invariance)	0.936	0.935	-0.004	-0.008	0.053 (0.047, 0.059)	0.055 (0.050, 0.061)
Model 3 (Strong invariance)	0.912	0.918	-0.024	-0.018	0.059 (0.053, 0.064)	0.059 (0.054, 0.065)

**Table 4:** Fit Indices Used for Assessing Factor Invariance Across NDD status and ICSEA level (*N*=835)

Model	CFI		∆CFI (vs. preceding model)		RMSEA (90% CI)	
	ICSEA	NDD	ICSEA	NDD	ICSEA	NDD
Model 1 (Unconstrained)	0.936	0.943	-	-	0.046 (0.041, 0.052)	0.053 (0.047, 0.059)
Model 2 (Weak invariance)	0.933	0.942	-0.003	-0.001	0.045 (0.040, 0.050)	0.051 (0.045, 0.057)
Model 3 (Strong invariance)	0.919	0.936	-0.014	-0.006	0.046 (0.042, 0.051)	0.051 (0.045, 0.056)

*ICSEA* Index of Community Socio-Educational Advantage, *NDD* Neurodevelopmental Disorder

**Table 5:** Means (Standard Deviations) for (i) Academic Success and the Future and (ii) Peer Relationships, shown by ICSEA level, NDD status, and Age

		Academic Success and the Future	Peer Relationships
ICSEA	Bottom 25% (n = 165)	3.17 (2.37)^a^	1.15 (1.31)^b^
	Middle 50% (n = 489)	3.13 (2.32)^a^	1.04 (1.25)^b^
	Top 25% (n = 181)	3.38 (2.08)^a^	1.10 (1.07)^b^
NDD status	NDD (n = 92)	2.14 (1.93)^c^	0.71 (0.70)^d^
	Non-NDD (n = 743)	3.32 (2.29)^c^	1.12 (1.27)^d^
Age	10-13 (n = 384)	2.91 (2.20)^e^	1.20 (1.29)^f^
	14+ (n = 449)	3.42 (2.33)^e^	0.97 (1.15)^f^
	*Overall* (N = 835)	3.19 (2.28)^g^	1.06 (1.23)^g^

*ICSEA* Index of Community Socio-Educational Advantage, *NDD* Neurodevelopmental Disorder

^a,b^Not significantly different (*p* > .05)

^d,e,f^Significantly different (*p* < .01)

^c,g^Significantly different (*p* < .001)

**Table 6:** Correlations^a^ between PAWS sub-scales and SDQ, PALS, and CDI2

	2.	3.	4.	5.	6.	7.	8.	9.	10.
1. Academic Future	0.50***	0.36***	0.00	0.41***	0.21***	0.31***	-0.20***	0.14***	0.21***
2. Peer Relationships	-	0.48***	0.06	0.51***	0.29***	0.46***	-0.36***	-0.01	0.39***
3. CDI:SR[S]2		-	-0.31***	0.70***	0.57***	0.61***	-0.56***	0.12**	0.31***
4. SDQ: Prosocial			-	-0.21***	-0.38***	-0.20***	0.35***	-0.13***	0.07*
5. SDQ: Internalising				-	0.46***	0.57***	-0.54***	0.15***	0.30***
6. SDQ: Externalising					-	0.30***	-0.31***	0.05	0.22***
7. PALS: Isolation						-	-0.70***	0.05	0.38***
8. PALS: Friendships							-	-0.08*	-0.21***
9. PALS: Positive Attitude								-	0.39***
10. PALS: Negative Attitude									-

*p < .05; **p < .01; ***p < .001

^a^Correlations based on sample sizes ranging from 818 to 835 dependent upon missing data.

*PAWS* Perth Adolescent Worries Scale, *CDI:SR[S]2* Children’s Depression Inventory: Self-Report (Short) 2 T-Score (Kovacs, 2004), *SDQ* Strengths and Difficulties Questionnaire (Goodman, 1997; Goodman et al., 2010), *PALS* Perth Aloneness Scale (Houghton et al., 2014)

## Supplementary Information

Below is the link to the electronic supplementary material.

Supplementary file1 (DOCX 63 KB)

Supplementary file2 (ZIP 91 KB)

## Data Availability

All data generated or analyzed during this study are included in this published article and its supplementary information files.
