# Supplementary material for: Development of the Perth Adolescent Worry Scale (PAWS)
Source: Res Child Adolesc Psychopathol. 2021 Aug 20;50(4):521–35. doi: 10.1007/s10802-021-00853-6 (PMC8940868; doi:10.1007/s10802-021-00853-6)
Supplement: Supplementary file 1 — Supplementary file1 (DOCX 63 KB) [file 10802_2021_853_MOESM1_ESM.docx]

Appendix

The Perth Adolescent Worry Scale.

Adolescents worry about a number of things and this is quite normal. Worrying is when you feel uneasy or are more than usually concerned about a situation or problem. Read the statements below and shade the circles that you think best apply to you.

For example, if you ‘often’ worry about your friends, you would shade in the circle for “often” and if you worried ‘a lot’ about your friends then you would shade the circle for “a lot”. Remember, shade in the circles below that best apply to you. Please answer all questions.

|  | How OFTEN do you worry about the following? | | | | When you worry, how MUCH do you worry? | | | |
| --- | --- | --- | --- | --- | --- | --- | --- | --- |
|  | Never | Sometimes | Often | Always | Not at all | A little bit | Somewhat | A lot |

Not doing well in school

Fitting in with other students

at school

Being bullied at school

Falling out with your friends

Your future

Being judged by your friends

People talking about you online

Keeping up with school work

What you will be doing when

you finish High school

Letting your parents down

Exams or tests

Having a boyfriend or girlfriend

(i.e. finding or maintaining

a relationship)

*Scoring instructions:*

Responses to each item should be recorded using 0 (Never/Not at all), 1 (Sometimes/ A little bit), 2 (Often/ Somewhat), 3 (Always/ A lot). The frequency (‘how OFTEN)’ and the degree (‘how MUCH’) responses for each item are then multiplied to create a single 0-9 score for all 12 items. These combined item scores are used to create weighted scale scores using the following formulae:

Academic Success and the Future scale: (‘Exams or tests’ * .126) + (‘Keep up with school work’ * .149) + (‘Not doing well in school’ * .179) + (‘Your future’ * .156) + (‘What you will be doing when you finish High school’ * .141) + (‘Letting your parents down * .119)

Peer Relationships scale: (‘People talking about you online’ * .107) + (‘Fitting in with other students at school’ * .076) + (‘Falling out with your friends’ * .105) + (‘Being judged by your friends’ * .179) + (‘Being bullied at school’ * .091) + (‘Having a boyfriend or girlfriend’ * .043)
